# Editorial: Rising stars in red blood cell physiology: 2022

**DOI:** 10.3389/fphys.2022.1020144

**Published:** 2022-09-09

**Authors:** Angelo D’Alessandro

**Affiliations:** Department of Biochemistry and Molecular Genetics, University of Colorado Denver—Anschutz Medical Campus, Aurora, CO, United States

**Keywords:** red blood cell, omics, biophysics, biochemistry, metabolism, transfusion

Red blood cells (RBCs) are the most abundant host cell in the human body, accounting for ∼83% of the total cells. While their role in gas transport has long been appreciated, many novel roles for mature RBCs in systems physiology and immunity (Lam et al., 2021) are rapidly emerging–a concept that has been crystallized in the provocative definition of RBCs as an “organ” (Nemkov et al., 2018). Assuming a mean cell volume of ∼87 fl, 25 trillion RBCs in an adult individual (∼5 L of blood at a five million/microliter RBCs) would occupy a total volume of ∼2.175 L. With most amino acid concentrations ranging from the low micromolar to hundreds of micromolar in a mature RBCs (Catala et al., 2018), RBCs represent at the very least a large circulating reservoir of nutrients of critical metabolic relevance in health and disease. In this view, it is interesting to note that the concept of metabolic reprogramming permeates most of contemporary research, from cancer to inflammation, from immunometabolism to aging, from exercise physiology to cardiovascular disease, emergency medicine, responses to physiological or pathological hypoxia (Hanahan and Weinberg, 2011; O'Neill et al., 2016; D'Alessandro et al., 2018; Drapela et al., 2022). However, little to no attention is paid to the RBC in most of this literature, with the mature erythrocyte often regarded as an inert bystander.

Concepts like mitochondrial dysfunction dominate the lexicon in most of current literature (Murphy and Hartley, 2018), while limited attention is paid to substrate availability (oxygen and metabolic substrates for mitochondrial metabolism) to the extent their distribution through the human body is regulated by RBC function. Indeed, while RBCs lack nuclei and organelles, and are thus incapable of *de novo* protein synthesis, they may compete for some key substrates (e.g., methionine), whose uptake and consumption is increased as a strategy to drive isoaspartyl-damage repair following oxidant insults (D'Alessandro et al., 2021). In so doing, stressed RBCs subtract free methionine from distal tissues that can otherwise consume the same molecules to fuel epigenetic regulation by mechanism of methylation of histones or nucleic acids (CpG islands, methyl-6-adenosine in DNA and RNA, respectively). While a growing body of literature is exploiting dietary interventions [from time-restricted feeding to starvation (Sanderson et al., 2019; Xie et al., 2022)], limited to no study has hitherto addressed the potential role of RBCs in epigenetic regulation in clinically relevant contexts.

On the other hand, a burgeoning literature has documented the life-saving role of RBC transfusion to millions of massively or chronically transfused recipients every year worldwide. RBC studies, including some included in the present Research Topic, have documented the effects of storage under blood bank conditions on RBC physiology and function (Yoshida et al., 2019). Tzounakas et al. here provide evidence that some of these so-called storage lesions are not entirely restored upon 24 h incubation of stored RBCs to recipient-mimicking conditions. Heterogeneity in genetic backgrounds impacts the quality of stored RBCs (Page et al., 2021) and transfusion outcomes (Roubinian et al., 2019). Here, Anastasiadi et al. expand on this concept, by providing evidence that background genetic heterogeneity modulates the redox storage lesion in RBCs donated by βThal-trait heterozygotes–who are eligible for blood donation.

Focusing on another hemoglobinopathy, Nemkov et al. describe the metabolic impact of sickle cell trait on systems metabolism. Subjects with sickle cell trait carry one copy of mutated β-globin gene at position E6V, which results in the production of sickle hemoglobin (HbS). Individuals with sickle cell trait are generally benign/asymptomatic, however carriers of such trait may develop certain adverse outcomes such as renal complications, venous thromboembolism, exercise-induced rhabdomyolysis. Here the authors show that systems metabolism–especially acyl-carnitines and carboxylic acids–are significantly dysregulated in these subjects. Alterations in these pathways significantly associated with renal and cardiovascular function, paving the way for similar studies in patients with sickle cell disease who inherited of two copies of the mutated gene. Alterations to the acyl-carnitine system has been recently associated with dysregulation of the Lands cycle that participates in the repairing of damaged RBC membranes in sickle cell disease (Wu et al., 2016) or following exercise-induced oxidant stress (Nemkov et al., 2021), or in chronic kidney disease (Bissinger et al., 2021; Xu et al., 2022). Similar alterations of lipid damage-repair mechanisms to the RBC membrane have been posited to play a role in mediating the severity of COVID-19 (Thomas et al., 2020). Expanding on this concept, Marchi et al. here report a systematic evaluation of RBCs morphology at peripheral blood smear in COVID-19 patients within the first 72 h from hospital admission. Through these analyses, they describe a variety of abnormalities in RBCs morphology was observed in 65% patients.

Of note, studies targeting RBC membrane composition are critical to inform novel in silico models of the erythrocyte cytoplasmic membrane–as summarized here by Himbert and Rheinstadter. The cytoplasmic membrane is expected to dominate the elastic behavior on small, nanometer length scales, which are most relevant for cellular processes that take place between the fibrils of the cytoskeleton. The mechanical properties of RBC membranes are intrinsically linked to the molecular composition and organization of their shell–and appear to be particularly influenced by the interaction between polyunsaturated lipids and cholesterol. The cytoplasmic membrane is expected to dominate the elastic behavior on small, nanometer length scales, while dysregulation of RBC membrane composition in response to pathological state such as those listed in the previous paragraph ultimately impacts the RBC capacity to squeeze through peripheral capillaries and exchange gas and nutrients in those regions.

Far from being comprehensive, this Research Topic of papers from talented, early-stage investigators wants to represent a token of appreciation and recognition towards the next generation of RBC enthusiasts, in the hope to expand this group in the years to come, through collaborative networks such as the one fostered by the community of contributors to the Red Blood Cell Physiology section of Frontiers in Physiology.
